# Prenatal diagnosis of 22q11.2 copy number abnormalities in fetuses via single nucleotide polymorphism array

**DOI:** 10.1007/s11033-020-05815-7

**Published:** 2020-09-15

**Authors:** Meiying Cai, Na Lin, Linjuan Su, Xiaoqing Wu, Xiaorui Xie, Ying Li, Yuan Lin, Hailong Huang, Liangpu Xu

**Affiliations:** Department of the Prenatal Diagnosis Center, Fujian Maternity and Child Health Hospital, Affiliated Hospital of Fujian Medical University, Fujian Key Laboratory for Prenatal Diagnosis and Birth Defect, Fuzhou, China

**Keywords:** 22q11.2 copy number abnormality, Single nucleotide polymorphism array, Prenatal diagnosis, Genomic diseases

## Abstract

The q11.2 region on chromosome 22 contains numerous low-copy repeats that lead to deleted or duplicated regions in the chromosome, thereby resulting in different syndromes characterized by intellectual disabilities or congenital anomalies. The association between patient phenotypes and 22q11.2 copy number abnormalities has been previously described in postnatal cases; however, these features have not been systematically evaluated in prenatal cases because of limitations in phenotypic identification in prenatal testing. In this study, we investigated the detection rate of 22q11.2 copy number abnormalities in 2500 fetuses using single nucleotide polymorphism (SNP) array and determined the common abnormal ultrasound findings in fetuses carrying the 22q11.2 copy number abnormalities. The 22q11.2 copy number abnormalities were identified in 13 fetuses with cardiovascular malformations (6/13), kidney malformations (3/13), isolated ultrasound markers (3/13), or high-risk Down syndrome based on maternal serum screening (1/13). Approximately 0.5% (13/2500) of the fetuses harbored 22q11.2 copy number abnormalities. The most frequent ultrasound findings in fetuses with these abnormalities were cardiovascular malformations, followed by kidney malformations and isolated ultrasound markers. Prenatal diagnosis of these genetic abnormalities allows for the delineation of differential diagnoses, characterization of a wide spectrum of associated malformations, and determination of associations that exist between prenatal diagnosis and obstetrical outcomes.

## Introduction

Chromosome 22 has long been implicated in genomic diseases, such as 22q11.2 deletion syndrome (22q11.2DS) and cat-eye syndrome (CES), which are associated with decreased and increased gene dosages, respectively [1]. Approximately 1 in every 4000 live births shows 22q11.2DS, also known as velocardiofacial or DiGeorge syndrome, making the most common microdeletion syndrome in humans [2]. The 22q11.2DS phenotype is highly variable with over 180 clinical features [3], including congenital conotruncal cardiac defects, characteristic facial features, immunodeficiency, palatal abnormalities, hypocalcemia, urogenital abnormalities, a spectrum of cognitive deficits, and psychiatric symptoms. On the contrary, CES is rare disorder caused by duplication of a part of chromosome 22 [4]. CES is characterized by preauricular tags or pits, ocular coloboma, dysmorphic facial features, and cardiac, urogenital, and anorectal anomalies [5].

The long arm (q region) of chromosome 22 is vulnerable to chromosomal rearrangement because of the large number of low-copy repeats (LCR) in this region, which mediate non-allelic homologous recombination resulting in unequal crossover rearrangements [6]. These mechanisms lead to deleted or duplicated regions of the chromosome, resulting in different syndromes characterized by intellectual disabilities and/or congenital anomalies [4]. More than 90% of the patients with 22q11.2DS have a classic 3 Mb deletion spanning LCR22A–D, including *TBX1 *[7]. A corresponding chromosomal duplication in 22q11.2 in LCR22A–D has also been described, which is associated with diverse phenotypic abnormalities. This duplication may result in phenotypes similar to those associated with 22q11.2DS; however, this also occurs in individuals with a normal phenotype [8–11]. Less than 10% of patients with either 22q11.2 deletions or duplications are affected in a smaller 1.5 Mb region spanning LCR22A–B [12–14].

The etiology in most patients is a classic 3 Mb recurrent deletion or duplication in 22q11.2. However, cases of atypical deletions and duplications with different sizes and locations have also been described, generally with a milder, slightly different phenotype for duplications; however, few studies have focused on 22q11.2 copy number abnormalities in fetuses. In this study, we report 13 cases with 22q11.2 copy number abnormalities (including atypical breakpoints), which were all diagnosed prenatally. In all cases, we delineated the differential diagnoses, illustrated the spectrum of associated malformations, and explored the association between prenatal diagnosis of 22q11.2 copy number abnormalities and obstetrical outcomes. Although few cases are included in this study, these results likely represent the intrauterine phenotypic profiles of 22q11.2 copy number abnormalities, thereby improving the understanding of intrauterine phenotypic heterogeneity associated with these abnormalities.

## Materials and methods

### Patient data

The study was conducted between January 2016 and December 2018 at the Prenatal Diagnosis Center of the Fujian Provincial Maternal and Children Health Hospital. A total of 2500 fetuses participated in this study, including 1821 fetuses with abnormal sonographic findings (classified as fetal structural malformations, ultrasound soft markers, hydrops fetalis, fetal growth restriction, and abnormal amniotic fluid volume), 432 fetuses with advanced maternal age (over the age of 35 years), and 247 fetuses with established high-risk Down syndrome based on maternal serum screening (cut-off for high-risk was 1:270). This study was approved by the ethics committee of Fujian Provincial Maternal and Child Health Hospital, and informed consent was obtained from the parents for invasive prenatal diagnosis. Amniotic fluid was collected by amniocentesis at 18–24 weeks of gestation and cord blood was collected at 24 weeks of gestation. The median gestational age for screening was 26 weeks (18–34 weeks).

### SNP array

Our laboratory previously established the SNP array technology that was used in this study [15]. Genomic DNA was directly extracted from uncultured amniotic fluid and cord blood samples using the QIAamp DNA Blood Mini kits (Qiagen, Hilden, Germany). The genome-wide high-resolution SNP array CytoScan HD (Affymetrix Genome CytoScan 750 K, Affymetrix, Santa Clara, CA, USA), including SNPs and oligonucleotide probes, was used in this study. DNA (250 ng) was amplified, labeled, and hybridized. The copy number variant (CNV) reporting filter was set at > 100 kb with a minimum set of 50 marker counts.The results were analyzed using Chromosome Analysis Suite software (Affymetrix) and annotated based on GRCh37 (hg19).The CNVs identified using the SNP array were classified as pathogenic (P), variants of uncertain significance (VOUS), and benign (B) according to the American College of Medical Genetics guidelines [16]. Pathogenic CNV and VOUS were classified as abnormal. All annotated CNVs were experimentally validated by fluorescence in situ hybridization.

## Results

Among 2500 fetuses subjected to SNP array analysis, 227 fetuses had abnormal CNVs. Among 1821 fetuses with abnormal sonographic findings, 165 fetuses had abnormal CNVs (9.1%, 165/1821). Among 432 fetuses with advanced maternal age, 26 fetuses had abnormal CNVs (6.0%, 26/432). Among 247 fetuses with established high-risk Down syndrome based on serum screening, 36 fetuses had abnormal CNVs (14.6%, 36/247). Among 227 fetuses with abnormal CNVs, the 22q11.2 copy number abnormalities were most common. The 22q11.2 copy number abnormalities were identified in 13 cases of 11 singleton pregnancies and 1 twin pregnancy, accounting for approximately 0.5% of the cases. Out of the 13 fetuses carrying 22q11.2 copy number abnormalities, seven were deletions and six were duplications, indicating cardiovascular malformations (6/13), kidney malformations (3/13), isolated ultrasound markers (3/13), or high-risk Down syndrome based on maternal serum screening (1/13). All parents were healthy and non-consanguineous.

### Molecular characterization of 22q11.2 deletion in fetuses

Five cases were found to harbor a classic 22q11.2 deletion that encompassed *TBX1*and contributed to 22q11.2DS. For instance, a 36-year-old mother in case E2351 was pregnant with her second child. The fetus in this case presented with a ventricular septal defect, right-sided aortic arch, U-shaped vascular ring, aberrant left subclavian artery, and classic 3.1 Mb deletion. Furthermore, a 35-year-old mother in case E2503 had two previous healthy children. A fetal echocardiography at 20 weeks of gestation revealed tetralogy of Fallot. SNP results also revealed a classic 3.1 Mb deletion. Another case, P4643, was a 31-year-old primigravid mother. At 18 weeks of gestation, ultrasound examination revealed ventricular septal defect and pulmonary atresia. SNP analysis revealed a classic 2.8 Mb deletion. Genetic analysis of the parents revealed normal results. In case G9924, a 32-year-old mother was pregnant with her second child. The mother’s elder child had congenital heart disease and physical retardation. Ultrasound anomalies were accompanied with bilateral choroid plexus cysts. SNP results revealed a classic 3.1 Mb deletion. Genetic analysis of the parents showed that case G9924 arose from a maternal deletion on chromosome 22. The mother’s elder child was also found to carry this deletion. Lastly, case G9932 was a 27-year-old gravida 2, para 1 mother with an unremarkable personal history. Ultrasound examination at 23 weeks of gestation revealed an ectopic right kidney with polycystic dysplasia. SNP array detected a classic 2.8 Mb deletion. Ultrasound results for case G9924 and case G9932 revealed no abnormalities in the heart.

The results also revealed two cases with atypical deletions. In case W23, a 30-year-old mother had one previous healthy child. The family history was found to be non-significant. However, maternal serum screening resulted in a high Down syndrome risk estimate of 1:135 and the attempts to cultivate amniotic fluid from this patient failed. The remaining amniotic fluid was subjected to SNP array, which showed an atypical 2.0 Mb deletion. Similarly, the patient in case P4666 was a 31-year-old primigravida. Ultrasound anomalies were accompanied with nuchal cystichygroma and increased nuchal translucency in the fetus at 19 weeks of gestation. SNP array analysis detected an atypical 1.0 Mb 22q11.21–q11.22 deletion. Genetic analysis of the parents was non-significant (Table 1).

**Table 1:** Detected 22q11 deletions in fetuses and the resulting phenotypes

Case	SNP results	Size (Mb)	Phenotype	Pathogenicity classification	Gene(s)	Inheritance	Postnatal outcome
E2351	Arr[hg19]22q11.21(18,648,855–21,800,471) × 1	3.1	Ventricular septal defect, right aortic arch, U-shaped vascular ring, aberrant left subclavian artery	P	DGCR6, DGCR2, DGCR14, TBX1, DGCR8, DGCR16	Unknown	TP
E2503	Arr[hg19]22q11.21(18,648,855–21,800,471) × 1	3.1	Tetralogy of Fallot	P	DGCR6, DGCR2, DGCR14, TBX1, DGCR8, DGCR16	Unknown	TP
G9924	Arr[hg19]22q11.21(18,648,855–21,800,471) × 1	3.1	Bilateral choroid plexus cyst	P	DGCR6, DGCR2, DGCR14, TBX1, DGCR8, DGCR16	Maternal	TP
G9932	Arr[hg19]22q11.21(18,916,842–21,800,471) × 1	2.8	Ectopic right kidney with polycystic dysplasia	P	DGCR6, DGCR2, DGCR14, TBX1, DGCR8, DGCR16	Unknown	TP
P4643	Arr[hg19]22q11.21(18,919,477–21,800,471) × 1	2.8	Ventricular septal defect, pulmonary atresia	P	DGCR6, DGCR2, DGCR14, TBX1, DGCR8, DGCR16	De novo	TP
W23	Arr[hg19]22q11.21(18,631,364–20,729,389) × 1	2.0	High risk of Down syndrome serum screening	P	DGCR6,DGCR2, DGCR14, TBX1, DGCR8, DGCR16	De novo	TP
P4666	Arr[hg19]22q11.21(20,716,876–21,800,471)	1.0	Nuchal cystic hygroma, increased nuchal translucency	VOUS	ZNF74, SCARF2, KLHL22, POM121L4P, TMEM191A, PI4KA, SERPIND1,SNAP29, CRKL, LOC101928891, AIFM3, LZTR1, THAP7, THAP7AS1, TUBA3FP, P2RX6, SLC7A4, MIR649, P2RX6P, LRRC74B, BCRP2, LOC102724728, FAM230B, POM121L8P, LOC100996335, RIMBP3C, RIMBP3B, HIC2	De novo	TP

*P* pathogenic, *TP* termination of pregnancy, *VOUS* variation of uncertain clinical significance, *SNP* single-nucleotide polymorphism

### Molecular characterization of 22q11.2 duplication in fetuses

Microduplicationin 22q11.2 was detected in six separate cases by SNP analysis. Herein, we describe cases E2151, G8565, and Z18, all of which showed a duplicationin 22q11.2 with few overlapping features common to22q11.2DS. The patient in case E2151 was a 31-year-old gravida 2, para 1 with an unremarkable personal history. Ultrasound examination at 31 weeks of gestation revealed a complex cardiac defect with oval valve aneurysm, a small ascending aorta, and aortic arch associated with poor prognosis. SNP results revealed a classic 3.1 Mb duplication. In addition, the 37-year-old mother in case G8565 had one previous healthy child and two miscarriages. The family history was non-significant. Ultrasound examination at 18 weeks of gestation revealed a complex cardiac defect with ventricular septal defect, aortic dysplasia, and enhanced ventricular echo. SNP array detected a classic 1.7 Mb duplication that resulted in CES. Lastly, the patient in case Z18 was a 39-year-old primigravida. At 24 weeks of gestation, ultrasound examination revealed ventricular septal defect, aberrant subclavian artery, double superior vena cava, and a single umbilical artery. SNP array also showed a classic 1.7 Mb duplication resulting in CES.

Cases P4876 and P4877 were unique as they were dizygotic twin fetuses. The 28-year-old mother had three previous miscarriages. The mother had not conceived for 2 years and, therefore, used in vitro fertilization and embryo transfer to achieve her current pregnancy with dizygotic twins. Ultrasound examination at 24 weeks of gestation revealed that both the dizygotic fetuses had unilateral polycystic kidney dysplasia. SNP array detected an atypical 1.0 Mb duplication. Genetic analysis of the parents revealed normal results (Table 2).

**Table 2:** Detected 22q11 duplications in fetuses and the resulting phenotypes

Case	SNP results	Size (Mb)	Phenotype	Pathogenicity classification	Inheritance	Postnatal outcome
E2151	Arr[hg19]22q11.21(18,649,189–21,800,471) × 3	3.1	Oval valve aneurysm, small ascending aorta, aortic arch	P	Unknown	TP
G8565	Arr[hg19]22q11.1q11.21(16,888,899–18,649,190) × 4	1.7	Ventricular septal defect, aortic dysplasia, enhanced ventricular echo	P	Unknown	TP
Z18	Arr[hg19]22q11.1q11.21(16,888,899–18,649,190) × 4	1.7	Ventricular septal defect, aberrant subclavian artery. double superior vena cava, Single umbilical artery	P	Unknown	TP
P4876	Arr[hg19]22q11.21(20,730,143–21,800,471) × 3	1.0	Unilateral polycystic kidney dysplasia	VOUS	De novo	TD
P4877	Arr[hg19]22q11.21(20,730,143–21,800,471) × 3	1.0	Unilateral polycystic kidney dysplasia	VOUS	De novo	TD
E3027	Arr[hg19]22q11.21(18,648,855–21,459,713) × 3	2.8	Increased nuchal translucency, FGR	VOUS	Maternal	TP

*FGR* fetal growth restriction, *P* pathogenic, *TD* term delivery, *TP* termination of pregnancy, *VOUS* variation of uncertain clinical significance, *SNP* single-nucleotide polymorphism

The patient in case E3027 was a 23-year-old primipara. Ultrasound examination revealed an increased nuchal translucency and fetal growth restrictionin the fetus at 30 weeks of gestation. SNP array analysis detected a 2.8 Mb 22q11.2 duplication. Genetic analysis of the parents showed that the variation was inherited from the unaffected mother.

### Inheritance analysis and obstetrical outcomes

Hereditary information was screened for seven families where in SNP array analysis showed 22q11.2 copy number abnormalities (parents from six cases refused the collection of peripheral venous blood). Parental analysis revealed that two of the fetuses had abnormalities inherited from an unaffected parent, while in the five remaining fetuses, the CNVs occurred de novo.

The decision to terminate 11 of the pregnancies, according to the mothers, was because of the presence of abnormal CNVs. However, the parents of the dizygotic twins continued to full-term delivery although the SNP results were VOUS.

## Discussion

SNP array is an important prenatal genetic diagnostic tool for exploring the genetic causes of fetuses with ultrasound abnormalities. In this study, out of the 2500 fetuses subjected to SNP array analysis, 227 fetuses had abnormal CNVs. Among 227 fetuses with abnormal CNVs, 165 fetuses with abnormal sonographic findings had abnormal CNVs (9.1%, 165/1821), 26 fetuses with advanced maternal age had abnormal CNVs (6.0%, 26/432), and 247 fetuses with established high-risk Down syndrome based on serum screening had abnormal CNVs (14.6%, 36/247). Out of the 227 fetuses with abnormal CNVs, 22q11.2 copy number abnormalities were most common. Thirteen fetuses had CNVs in chromosome 22 but showed normal karyotype analysis results. Thus, SNP array is superior to karyotype analysis because of its ability to detect CNVs with high accuracy and resolution. Amniotic fluid cell culture was unsuccessful in one case and could not be analyzed further; however, SNP array detected genetic anomalies.

The primary causes of chromosome 22 abnormalities are LCR-mediated non-allelic homologous disorders and unequal intra- or inter-chromosomal recombination during meiosis [17]. In addition to the classic deletion/replication region (3 Mb) at the proximal and distal breakpoints, several atypical deletions or replications with variable breakpoints can be caused by different recombinants mediated by LCR22A and LCR22H. In this study, 22q11.2 copy number abnormalities in 13 cases were identified. Seven of these abnormalities were deletions and six were duplications. Nine cases exhibited classic deletion/replication within this region,while four cases were atypical with variable breakpoints likely caused by different recombinants mediated by LCR22A–H.

Our study revealed that cases E2351, E2503, and G9924 shared a common 3.1 Mb hemizygous 22q11.2 deletion, which is consistent with the results in the literature [18]. The sonogram findings for cases E2351 and E2503 revealed cardiac abnormalities; however, case G9924 showed only bilateral choroid plexus cysts. Additionally, case P4643 was characterized by a ventricular septal defect and pulmonary atresia and case G9932 by an ectopic right kidney with polycystic dysplasia. Both cases were accompanied by a 2.8 Mb deletion. These results demonstrate highly variable abnormalities in the heart, which is consistent with the highly variable phenotype associated with 22q11.2DS. Ultrasound technology may be inefficient in effectively screening the abnormalities and it is possible that the varying size of the deletions contributed to the variable phenotypes found to be associated with the 22q11.2 deletion cases [19].

Interestingly, in case W23, maternal serum screening revealed a high-risk estimate of 1:135 for Down syndrome; although analysis of the amniotic cell culture was unsuccessful, SNP analysis of the remaining amniotic fluid detected a 2.0 Mb deletion. Case P4666 was determined to have an atypical 1.0 Mb deletion of 22q11.21–q11.22 accompanied by a nuchal cystichygroma and increased nuchal translucency. Notably, genetic analysis of the parents showed normal results. According to the database, although this region is associated with a 22q11.2 deletion, the clinical significance is unclear.

Recently, new cases of 22q11.2 duplication have been described [20, 21]. In our study, six 22q11.2 duplications were identified via abnormal ultrasound. Cases E2151 and E3027 were identified with 3.0 and 2.8 Mb duplications, respectively, corresponding in size to the classical deletions observed in patients with 22q11.2DS. We also found that cases G8565 and Z18 had a tetrasomy of 22q11.1q11.21that included the proximal flanking region of chromosome 22, which is associated with CES [5, 22]. Cases P4876 and P4877 were dizygotic twin fetuses with unilateral polycystic kidney dysplasia accompanied by an atypical 1.0 Mb duplication. The region in which the duplication occurred includes 29 Online Mendelian Inheritance in Man genes. According to the database, although this region is associated with a 22q11.2 duplication, the clinical significance is unclear.

Out of the 22q11.2 copy number abnormalities present in the 13 cases, five cases were de novo, two cases were inherited from unaffected mothers, and six cases had an unknown inheritance because parental specimens were unavailable. Parental analysis showed that fetus G9924 inherited a maternal 22q11.2 deletion; the mother’s elder child, who also had congenital heart disease, showed an identical genetic abnormality. Interestingly, the phenotype of the mother was normal; however, an abnormal ultrasound indicated choroid plexus cysts in the fetus. The mother’s elder child had syndromic congenital heart disease. Clinical manifestations of 22q11.2DS vary significantly even within a single family and are not definitively associated with a single genotype. The parents were informed that the infant may show symptoms of disease, leading to their decision to terminate the pregnancy. Parental analysis determined that fetus E3027 inherited a maternal 22q11.2 duplication. The mother was a healthy 23-year-old primipara. Although, according to the database, the clinical significance of this region associated with a 22q11.2 duplication is unclear, the parents decided to terminate the pregnancy. The parents of case P4666 also terminated the pregnancy when genetic analysis revealed the presence of VOUS. Based on the informed decisions, 11 parents opted to terminate their pregnancies. Alternatively, the parents of the dizygotic twin fetuses continued the pregnancy to full term even after the SNP array was VOUS because they were unable to conceive and opted for in vitro fertilization and embryo transfer to achieve a successful pregnancy.

Common phenotypes associated with 22q11.2 deletion/duplication syndrome include congenital cardiac defects, characteristic facial appearance, immune deficiency, palatal abnormalities, hypocalcemia, urogenital abnormalities, and a varying degree of cognitive deficits and intellectual disability [23, 24]. However, our fetal ultrasound results presented variable abnormalities with few normal results. We suggest that any hypothetical correlation between the abnormal fetus and size and location of the 22q11.2 alterations may be masked by other genetic or epigenetic modifying factors. SNP technology identified atypical breakpoints that do not correlate with known regions, thereby suggesting a greater level of genomic complexity underlying the 22q11.2 region.

## Conclusions

We prenatally diagnosed 13 cases, among 2500 fetuses, with 22q11.2 copy number abnormalities. Prenatal analysis via SNP array allows for effective diagnosis of genetic abnormalities, delineation of differential diagnoses, characterization of a wide spectrum of associated malformations, and determination of the associations that exist between prenatal diagnosis and obstetrical outcomes. Approximately 0.5% of the fetuses harbored 22q11.2 copy number abnormalities. The most frequent ultrasound findings in fetuses with these abnormalities were cardiovascular malformations, followed by kidney malformations and isolated ultrasound markers.
